# Shaping Outcomes: Levodopa–Carbidopa Intestinal Gel Treatment and Nutrition in Parkinson’s Disease—A Prospective Observational Cohort Study

**DOI:** 10.3390/jcm14072321

**Published:** 2025-03-28

**Authors:** Monika Figura, Iwona Chaberska, Łukasz Milanowski, Magdalena Milewska, Dariusz Koziorowski

**Affiliations:** 1 Department of Neurology, Faculty of Health Sciences, Medical University of Warsaw, Kondratowicza 8 Street, 03-242 Warsaw, Poland; iwona1chaberska@gmail.com (I.C.); lukasz.milanowski@wum.edu.pl (Ł.M.); dariusz.koziorowski@wum.edu.pl (D.K.); 2 Department of Clinical Dietetics, Faculty of Health Sciences, Medical University of Warsaw, Ciołka 27 Street, 01-445 Warsaw, Poland; magdalena.milewska@wum.edu.pl

**Keywords:** levodopa–carbidopa intestinal gel, bioimpedance, nutrition, Parkinson’s disease

## Abstract

**Background/Objectives**: Parkinson’s Disease (PD) is a neurodegenerative disorder resulting in bradykinesia, rigidity and tremor, as well as numerous non-motor symptoms. Malnutrition in PD is correlated with levodopa-induced dyskinesia, decreased food intake, gastrointestinal symptoms and neurodegenerative processes. With disease progression, oral levodopa treatment becomes insufficient. One of the therapies used in advanced PD is levodopa–carbidopa intestinal gel. Its effect on the weight and nutrition of PD patients is poorly understood. The aim of this prospective single-center observational cohort study was to assess the effect of this treatment on weight, body composition and biochemical parameter changes over a two-year-long observation. The mood, cognition and motor status of the patients were also assessed. **Methods**: This study included 15 patients with advanced PD treated with levodopa–carbidopa intestinal gel. Body composition analysis, anthropometric measurements, blood tests, psychological assessments and disease control measurements were carried out over a span of two years after the initiation of therapy. **Results**: Significant improvement in disease management was observed. Anthropometric measurements, biochemical parameters and psychological assessments did not show significant differences. Among the body composition parameters, only resting metabolic rate and extracellular and intracellular water percentages were significantly affected. **Conclusions**: Our findings indicate a lack of negative effects of levodopa–carbidopa intestinal gel treatment on weight loss in patients with Parkinson’s Disease in a 2-year long observation period. Furthermore, better disease management may result in a lower energy expenditure due to less time with dyskinesia. The limitations of our study include a small study group and limited follow-up.

## 1. Introduction

Parkinson’s Disease (PD) is a progressive neurodegenerative disorder involving numerous motor and non-motor manifestations. It affects about 1–2 in 1000 people, including about 1% of the population above 60 years of age [1]. In Poland, its epidemiology is estimated at 269 per 100,000 people [2]. Interestingly, the prevalence is 1.2 times higher in women in Poland, while globally, it is estimated to be 1.4 times higher in men [2,3]. The acknowledged risk factors include older age, male sex, genetic predispositions and low socioeconomic status [3]. The clinical diagnosis of PD is based on presence of motor symptoms: bradykinesia combined with tremor and/or rigidity [4]. Numerous non-motor symptoms, including olfactory loss and cardiac denervation, may support this diagnosis [4]. People with Parkinson’s Disease (PwP) have a lower body weight than healthy controls [5]. Malnutrition in PD is a complex matter, with reduced nutritional intake, increased energy expenditure and central neurodegeneration being possible factors [5].

The definition of advanced PD varies. The 5-2-1 criteria, including 5 doses of levodopa per day, 2 h in an OFF state and 1 h of levodopa-induced dyskinesia (LID) per day are commonly applied [6]. Device-assisted therapies are beneficial for patients with advanced PD when the optimization of oral treatments has been exhausted [7]. Data from randomized trials unequivocally indicate that levodopa–carbidopa intestinal gel (LCIG) is effective in achieving the significant reduction in OFF time as well as troublesome dyskinesia in PD patients with motor complications with oral dopaminergic treatment [8]. The results from the COSMOS and GLORIA studies confirm these findings in long-term observations [9,10]. The Barcelona registry results indicate that the change in time of LID may vary depending on the duration of LCIG treatment [11]. Non-motor symptoms, which may be significantly improved by LCIG, include sleep, impulse control disorders and gastrointestinal and cardiovascular symptoms, among others [9,10]. This is also reflected by an improved quality of life of treated patients, namely a reduction in the PDQ-8 and PDQ-39 scores [9,10].

While advanced therapies are of vast benefit to PwP, there are limitations to each of the therapies. Device and gastrostomy-related complications are common [8]. Weight loss and malnutrition are commonly observed in the course of PD [12]. A variety of factors may contribute to these complications. These include the intensity of motor symptoms, such as rigidity, tremor and levodopa-induced dyskinesia, which are energy-consuming for patients. Non-motor symptoms—hyposmia, dysphagia, constipation, depression and dementia—may also contribute to this problem. Data from the GLORIA registry indicate that weight loss occurred in 6.7% of treated patients [10]. Interestingly, in a paper by Umemoto et al. comparing deep brain stimulation, LCIG and oral dopaminergic treatment, the authors did not report differences in weight loss between LCIG and oral treatment [13]. Only deep brain stimulation was noted as causing significantly less weight loss than oral treatment. In a paper by Fabbri et al., the authors identified time spent in LID as the main contributor to weight loss [14].

Bioimpedance measurement is a method of assessing body fluid volumes. It is based on measurements of resistance to a high-frequency, low-amplitude alternating electric current. It allows for a rapid, non-invasive and inexpensive evaluation of hydration and nutrition status [15].

The aim of our study was to assess how treatment with LCIG influenced body composition, weight and biochemical markers of nutrition. To answer this question, we performed a battery of tests, including anthropometric and bioimpedance analysis (BIA) measurements, blood tests and assessments of mood, cognition and motor status of PwP qualified for LCIG therapy. Our study provided a detailed insight into the exact mechanisms affecting weight and body composition in PwP treated with LCIG during an initial 2 years of therapy. It raised unique clinical implications for this group of patients.

## 2. Materials and Methods

### 2.1. Materials

Participants were recruited between December 2017 and March 2020 in the Department of Neurology, Faculty of Health Sciences, Medical University of Warsaw, Poland. Data were collected between March 2018 and February 2022. During this time, 15 patients with a diagnosis of advanced PD were included in this study. The main inclusion criteria for our study were providing consent to participate in the study, having a diagnosis of PD according to MDS criteria and undergoing treatment with LCIG [4]. The official criteria for qualification to LCIG in the years 2018–2022 in Poland included contraindications to deep brain stimulation, a diagnosis of PD for more than 5 years and the manifestation of motor complications from levodopa treatment (dyskinesia, wearing off of symptoms resulting in OFF periods), which were present for over 50% of the day according to Hauser diaries, and achieved with an optimized oral treatment regimen (at least 2 oral treatments with different mechanisms of action) [16].

The exclusion criteria for LCIG included dementia and pronounced psychotic symptoms; the lack of a caregiver to assist in pump management; suspicion of melanoma, narrow angle glaucoma, heart, renal or hepatic failure; acute stroke; comorbidities in which the administration of adrenergic agonists would be contraindicated and a lack of transluminescence in the abdominal wall during PEG implantation. Additional exclusion criteria included contraindications to BIA measurements—epilepsy, the presence of any kind of electric stimulators and pacemakers in the body (including DBS) and the presence of metallic objects in the body (joint prostheses, etc., with the exception of dental implants), massive skin lesions and massive dyskinesia during analysis.

**Figure 1:**
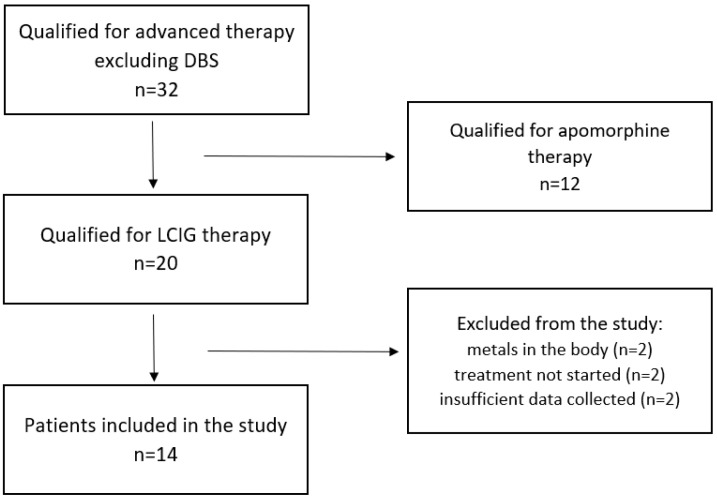
Flow diagram describing the process of patients’ inclusion in the study. DBS—deep brain stimulation, LCIG—levodopa–carbidopa intestinal gel.

Table 1 summarizes the clinical data of the PwP included in the study, while Figure 1 describes recruitment process of the patients included in the study, with reasons for exclusion.

The most common comorbidities were hypertension (5 participants), dyslipidemia (5), cervical (3) or lumbar (2) disc disorders, a past history of orthopedic surgery (3), a past history of gynecological surgery (2), diabetes mellitus (2) and gastritis (2). One patient had an initial BMI < 18.5 kg/m^2^, considered to be underweight. The dysphagia limit was assessed on admission in 13 patients and was above 20 mL in 7/13 patients, 15 mL in 3/13 and 5–10 mL in 3 patients. LCIG was administered for 16 h per day with night breaks, during which the patients were allowed to take additional doses of oral levodopa if needed. None of the patients received professional nutritional treatment during the period of observation.

The study protocol for this research was chosen and validated through a process designed to address a specific gap in the knowledge regarding the effects of LCIG treatment on nutritional parameters in PwP. The validation of the study protocol was based on a review of the existing literature and a recognition of the gaps in current research on nutrition and body composition in advanced PD. The choice of a prospective, single-center, observational cohort study design was deemed appropriate for observing changes over time in a clinical setting. The use of various measurement tools—such as BIA, anthropometric measurements, blood tests and psychological assessments—ensured that the study captured a broad range of health parameters to better understand the effects of LCIG treatment. Apart from the aforementioned inclusion and exclusion criteria, no other selection criteria were applied for the participants, so as to provide unbiased data. The study sample size was restricted by the number of PD patients qualified for LCIG treatment in our faculty during the recruitment period. This study has been approved by the Ethics Committee of the Medical University of Warsaw (KB/222/2018) and has therefore been performed in accordance with the ethical standards laid down in the Declaration of Helsinki of 1964 and its later amendments. All participants signed an informed consent form prior to their inclusion in the LCIG treatment program.

### 2.2. BIA Body Composition Analysis and Other Tests

Assessments were performed before (baseline, V0), 6 months (V0.5), 12 ± 3 months (V1) and 24 ± 3 months (V2) after the initiation of LCIG therapy. Psychological assessments were performed on an annual basis (baseline—V0, 12 months—V1 and 24 months—V2).

Body composition analysis was performed using the bioelectrical impedance analysis (BIA) method with the use of the Maltron Bioscan 920-II Multi-frequency Analyser (Maltron international, United Kingdom) in the Department of Neurology, Mazovian Brodno Hospital. The current parameters were an intensity of 800 μA at one (50 kHz) or multiple frequencies (5, 50, 100 and 200 kHz). The analysis was performed without intravenous fluid administration, in a supine position, with the limbs abducted from the body axis by 30 degrees. The electrodes were applied 5 cm apart on the dorsal central part of the hand between the carpal joint and the 3rd metacarpophalangeal joint and on the dorsal central part of the foot between the tarsal joint and the 3rd metatarsophalangeal joint, 5 cm apart. All measurements were performed in the same room, with uniform humidity and temperature. The study participants were supposed to meet the conditions of the guidelines concerning the body composition measurements issued by the European Society for Clinical Nutrition and Metabolism (ESPEN) [17,18]. On the day of the examination, the patients had to rest in a sitting position for about an hour before the measurements, to refrain from eating and drinking for 2–3 h prior to the test, to void urine 30 min before the test, to refrain from physical activity for 12 h prior to the test and to refrain from the consumption of alcohol, tea or caffeinated beverages for 24 h prior to the test.

The data obtained during measurements included the body mass index (BMI kg/m^2^); percentage of fat mass in the body (FAT%); the fat mass (FAT kg); fat-free mass (FFM kg, %); body cell mass (BCM, kg), which accounts mainly for the mass of the organs and muscles without adipose tissue; malnutrition index (MI); percentage of total body water (TBW%); intracellular water (ICW, l) and extracellular water (ECW, l) in the absolute weight and percentage of the total weight; the ECW to ICW ratio; the calculated resting metabolic rate (RMR kcal) and muscle mass (MW). Reference ranges are shown in Supplementary Table S1. Levodopa equivalent daily dose (LEDD) calculations were performed using a levodopa equivalent dose calculator available online [19].

Weight, waist and hip circumferences, abdominal skinfold and forearm skinfold thicknesses were obtained before BIA.

Blood samples for laboratory measurements were collected at baseline, after 6 months (V0.5) and after 1–2 years (V1) visits in the fasting state using venipuncture. The measurements performed included alanine transaminase (ALT), aspartate transaminase (AST), creatinine, total blood protein, albumin level, vitamin B12 level, low-density lipoprotein (LDL), high-density lipoprotein (HDL) and total cholesterol (TC) and triglicerydes (TG). Skinfold thickness was measured using a caliper on the arm (bicep area) and in the suprailiac area of the abdomen.

The prevalence of OFF states and LID were assessed with Hauser diaries at baseline (V0), after 6 months (V0.5), after 12 ± 3 months (V1) and after 24 ± 3 months (V2), filled out by the patients at home.

Psychological assessment included Addenbrooke’s Cognitive Examination-III (ACE-III) and Beck’s Depression Inventory, assessed in the Department of Neurology, Mazovian Brodno Hospital.

### 2.3. Statistical Analysis

The statistical analyses were performed using STATISTICA ver 13.5 software. The normality of the distribution was assessed using the Shapiro–Wilk test. As the data did not have a normal distribution, non-parametric tests were applied. Non-parametric variables are presented as the median and interquartile range (IQR). The repeated measures were assessed with the Friedman’s ANOVA test. The Dunn–Bonferroni test was performed for post hoc analysis. A statistical expansion was performed for the selected series of values in the bioimpedance measurements to fill out the missing data. The consecutive values of the series were extrapolated with the linear regression model, in which the independent variable was the number of observations in the series.

## 3. Results

Due to the complexity of the procedure, some randomly missing BIA values had to be replaced using the statistical method described above. This included the extrapolation of the baseline measurements (*n* = 1), and the V0.5 (*n* = 4), V1(*n* = 5) and V2 (*n* = 6) measurements. After the extrapolation of the randomly missing data, we observed significant differences regarding RMR between V0 and V1, and ECW% and ICW% between V0.5 and V1. We also observed significant changes in the ECW/ICW ratio between the baseline and the V1 measurement. No significant differences were observed regarding FAT, FAT kg, FFM kg, FFM%, BCM, MI, TBW, absolute weight of ICW and ECW and the MW. All the results of the BIA measurements are summarized in Table 2 and the post hoc analysis results for the significant differences are presented in Table 3.

Regarding patients’ motor status, we observed a significant reduction in the OFF time between V0 and visits V0.5 and V1. Similarly, we observed a significant reduction in time spent in troublesome dyskinesia between baseline (V0) and V2. This was accompanied by a significant increase in ON time, which was significantly longer in V0.5, V1 and V2 than at the baseline. No significant change in LEDD was observed. These results are summarized in Table 4 and Table 5.

The anthropometric measurements included BMI, weight, hip circumference, abdominal skinfold and forearm skinfold thickness. There were no significant differences observed during the 2-year-long observation (assessed at V0, V0.5, V1 and V2). These are presented in Table 4.

No significant differences in patients’ weight, hip circumference, abdominal skinfold and tricep skinfold thickness were observed during the 2-year-long observation. These are presented in Table 4.

Regarding the biochemical parameters connected with nutrition, an analysis comparing V0, V0.5 and V1 was performed using Friedman’s ANOVA. No statistically significant change was observed regarding total protein concentration, albumin, AST, vitamin B12, total cholesterol, high-density lipoprotein, low-density lipoprotein and triglycerides. The ALT activity and creatinine concentration varied significantly, but the mean results were within the normal range for all patients for the whole duration of the study. Additionally, no significant differences in depression symptoms (Beck’s Depression Inventory) and cognitive functions (Addenbrooke’s Cognitive Examination-III) were observed throughout the duration of the study at visits V0, V1 and V2. All results of the clinical and biochemical measurements are summarized in Table 4. Post hoc analysis results for significant differences are presented in Table 5.

The target fat percentage, water percentage and weight minima and maxima were obtained for each patient during BIA. Six patients with fat percentages higher or within the margin of the target fat percentage at V0 showed a fat percentage lower than the target at the end of the study. Only one of those subjects suffered from a weight below the target weight minimum at any point of the study. Three more patients had a fat percentage and weight lower than their target minimum for the whole study duration. No significant changes were present in water percentage compared with the target minimum and maximum.

## 4. Discussion

Weight loss has an important impact on prognosis for PwP. In a large observational study by Wills et al., the authors reported that PwP who experience weight loss during the disease duration have an increase in their UPDRS III score compared to PwP with a stable weight in a similar time period [20].

Song et al. studied the decrease in total body mass and changes in body composition prior to and a few years after PD diagnosis. The participants showed a trend in the loss of total body mass, fat percentage and fat mass 6–7 years before diagnosis, reaching significance 3–5 years into disease progression [21]. There were no significant changes observed in lean body mass. This is in accordance with the results obtained, as there were no significant changes in fat percentage and total body mass, although the number of subjects with a fat percentage below their target minimum tended to increase over the 2-year-long observation.

Similarly to other studies assessing the efficacy of LCIG treatment, we observed a significant reduction in the OFF time and the time spend with dyskinesia, as well as a significant increase in ON time in our cohort [9,10]. Interestingly, despite previous reports on weight loss in PD patients treated with LCIG, we did not report any significant weight loss in our cohort during a 2-year-long follow-up. This finding was confirmed by there being no significant differences in the total protein and albumin levels in peripheral blood measurements. A paper by Fabbri reported weight loss in approximately 60% of patients treated with LCIG [14]. That observation was longer, however, with a mean treatment time of 51.6 ± 28.5 months. The authors indicated that weight loss in LCIG-treated PwP correlates strongly with dyskinesia. This is in line with our findings, as dyskinesia was significantly reduced after pump treatment initiation in our cohort. This could be an important factor preventing excessive weight loss. The authors also raised some other important practical aspects regarding the management of PwP treated with LCIG. These included monitoring for dysphagia and weight loss in advanced PwP [14]. They also mentioned adjustments of LCIG flow to prevent dyskinesia and to reflect stable LEDD/kg in patients who experienced weight loss through the course of the disease.

Regarding the results of the bioimpedance measurements, we observed significant differences in RMR between measurements. Namely, we observed an increase in RMR between the baseline and the 12-month measurements. RMR at 24 months was also higher, although not significantly. Barichella et al. compared the resting energy expenditure between healthy people (HCs) and PwP, and concluded that there was no significant difference recorded [22]. This was, however, not the case for the more advanced PD group with a Hoehn and Yahr stage above three, where resting energy expenditure was significantly higher among PwP than HCs (1473 ± 347 vs. 1297 ± 187, respectively) [22]. In our cohort, an increase in RMR could have been related to an improvement in mobility and a need for higher energy intake, as opposed to a previously sedentary lifestyle, reflected by the significant improvement in ON time in the Hauser diaries [23]. As indirect calorimetry and not BIA is considered the gold standard for RMR assessment, the results obtained using BIA should be approached with caution [24].

We observed a decrease in extracellular water and an increase in intracellular water. No PwP was affected by clinically visible edema at any point of our study. To our knowledge, such changes have not been reported before, though their significance remains obscure. No significant changes or trends were observed regarding other parameters.

We observed no significant drop in vitamin B12 levels. There was, however, a tendency for lower levels of B12 in later observations. Patients treated with LCIG may have had an increased risk of B6, B12 and folate depletion. This may have been caused by a high dosage of levodopa administered continuously, as well as by the properties of the gel itself [25]. Regular control and diet intervention or supplementation should be conducted when vitamin depletion is seen.

We hypothesize that the presence of a caregiver could be an important factor contributing to the stability of weight during the observation. All patients included in the study were living with a caregiver (usually a spouse) and none were a nursing home resident, which is an important social contributor to malnutrition [26]. Therefore, even underweight patients at the beginning of the treatment were prevented from further weight loss with family care. Nevertheless, we acknowledge that professional nutritional treatment may be required in patients treated with LCIG for a longer time.

The main limitation of our study is the low sample size and the significant number of missed visits. This is, however, understandable, given the advanced PD status of the participants as well as the ongoing COVID-19 pandemic during the collection of the data. It led to serious limitations in the ability to recruit and perform BIA measurements in patients at all of the planned study points, as on-site visits were not always possible. The relatively small study sample may hinder results from reaching statistical significance. This is explained by our limited access to PD patients receiving LCIG treatment. Variables which are not taken into consideration are concomitant non-PD medications, such as antidepressant treatment, which may influence patients’ appetite and weight. While no patient received any specific nutritional interventions ordered by a dietician or nutrition specialist, we did not include patients’ specific dietary habits as a variable. The follow-up period is another limitation. While a 2-year-long observation is a significant time period, longer treatment (>5 years, clinical observation) with LCIG leads to visible malnutrition in PD patients. The extent of the impact of continuous treatment with LCIG via gastrostomy, as well as the impact on disease progression during this process, should be examined in detail. We did not include information on PD subtype (tremor dominant vs. postural instability–gait difficulty) as well as motor symptom laterality, which can influence the quality of life and level of motor impairment [27,28].

The external validity of this study is considered to be strong. The sample characteristics align closely with the general profile of patients treated with LCIG at other centers, particularly in terms of disease duration, motor complication status and other relevant factors. Although the study was conducted at a single center, the patients were referred from various locations across Poland, thus capturing a broad spectrum of socioeconomic backgrounds that may have influenced nutritional status. The intervention (LCIG) and the assessment modalities used (UPDRS, Hauser diaries, ACE, BDI questionnaires, blood assessments and BIA conducted with Maltron equipment) are well established, widely utilized methods with robust documentation in the scientific literature. A similar study design was employed in the research by Fabbri et al. [14], albeit with a larger sample size (44 patients).

A notable strength of our study lies in the more comprehensive assessment of nutritional status, providing a more detailed evaluation compared to previous studies.

In future research, the inclusion of GLIM criteria for the diagnosis of malnutrition should be considered [29]. These consist of three phenotypic and two etiologic criteria, the former being weight loss, body mass index and reduced muscle mass and the latter being reduced food intake or assimilation and disease burden/inflammation. In our study, none of the phenotypic criteria changed significantly. Patients were affected by diseases that could have mild-to-moderate effects on their nutrition, such as diabetes, hypertension and gastritis. All patients had a percutaneous endoscopic gastrostomy placed. Furthermore, PD may cause symptoms such as constipation, dysphagia, delayed gastric emptying, bradykinesia, depression and impaired cognitive function, which adversely affect food intake [30]. LID may result in a greater energy expenditure adding to disease burden. Thus, improvement in PD’s treatment thanks to LCIG would have a positive effect on weight as well as the motor status of patients.

## 5. Conclusions

To conclude, we did not observe clinically significant changes in body composition nor other markers of nutrition in our PwP cohort treated with LCIG for a 2-year-long period. Despite a general belief that LCIG treatment may induce significant weight loss, we did not observe a major deterioration in this regard. While our results should be interpreted carefully due to the aforementioned limitations, we believe a reduction in the time spent in LID is a key factor in weight loss management in advanced PD. The introduction of a new mode of administration—subcutaneous foslevodopa with foscarbidopa—will provide interesting new insights into the mechanisms of weight loss in PD and will hopefully allow for the reduction in this side effect. PwP should be constantly monitored for the symptoms of malnutrition on an individual basis and interventions should be applied for those who are underweight and in high-risk groups.

## Figures and Tables

**Table 1:** General characteristics of the study group at admission (*n* = 15). SD—standard deviation; UPRDS—Unified Parkinson’s Disease Rating Scale; DA—dopamine agonist; AChI—acetylcholinesterase inhibitor.

Feature	Mean ± SD
Age (years)	65.13 ± 7.09
Sex (male/female)	8/7
Body Mass Index (kg/m^2^)	24.66 ± 4.62
Disease Duration (years)	13.53 ± 3.93
L-dopa Equivalent Daily Dose (mg)	1533.21 ± 425.86
UPDRS Part III “OFF” (score)	51.53 ± 11.63
UPDRS Part III “ON” (score)	13.67 ± 5.34
Concomitant DA	6
(Number of people)	
Concomitant Amantadine	5
(Number of people)	
Concomitant AChI	2
(Number of people)	
Concomitant MAO-B Inhibitors	1

**Table 2:** The impact of the levodopa–carbidopa intestinal gel on the bioimpedance measurements after the statistical extrapolation of values. *p* is considered significant with values < 0.05.

Variable	Friedman’s ANOVA Test Value	*p*-Value	Median (IQR) V0	Median (IQR) V0.5	Median (IQR) V1	Median (IQR) V2
Resting Metabolic Rate (kcal)	8.31	0.04	1441.5 (1299–1612)	1412 (1312–1606)	1458 (1276–1606.5)	1458 (1276–1659)
Fat-Free Mass (kg)	5.41	0.14	48.78 (39.75–57.2)	48.31 (40.68–57.6)	46.44 (41.63–58.05)	48.84 (41.63–55.87)
Fat-Free Mass (%)	1.19	0.76	71.96 (66.77–75.42)	74.80 (67.30–80.35)	75.91 (69.31–79.69)	76.19 (73.07–80.61)
Fat Mass (kg)	0.56	0.91	48.78 (39.75–57.20)	48.31 (40.68–57.60)	46.44 (41.63–58.05)	48.84 (41.63–55.87)
Fat Mass (%)	0.82	0.84	71.96 (66.77–75.42)	74.8 (67.30–80.35)	75.91 (69.31–79.69)	76.19 (73.07–80.61)
Total Body Water (l)	3.8	0.28	19.57 (14.49–24.43)	17.86 (9.62–27.32)	17.38 (9.76–24.84)	14.21 (11.53–20.56)
Total Body Water (%)	0.89	0.83	28.05 (24.58–33.23)	25.21 (19.65–32.70)	24.09 (20.31–30.69)	23.81 (19.39–26.93)
Extracellular Water (l)	7.18	0.07	36.82 (25.35–42.94)	36.00 (28.48–43.48)	36.12 (28.90–42.68)	36.39 (28.90–42.31)
Extracellular Water (%)	12.07	0.01	47.03 (45.73–47.85)	47.01 (46.50–48.60)	46.08 (43.94–48.13)	46.21 (44.88–49.04)
Intracellular Water (l)	5.54	0.14	19.00 (15.20–22.97)	18.90 (15.92–23.05)	19.05 (16.24–22.55)	19.74 (16.24–22.55)
Intracellular Water (%)	12.07	0.001	19.00 (15.20–22.97)	18.90 (15.92–23.05)	19.05 (16.24–22.55)	19.74 (16.24–22.55)
Extracellular Water/Intracellular Water	12.41	0.01	0.89 (0.84–0.92)	0.89 (0.87–0.95)	0.85 (0.78–0.93)	0.86 (0.81–0.96)
Body Cell Mass (kg)	2.85	0.41	26.73 (21.09–31.07)	25.22 (23.12–30.95)	25.73 (23.75–31.14)	26.82 (23.75–31.14)
Muscle Mass (kg)	2.56	0.46	23.32 (18.47–26.67)	21.96 (20.24–26.67)	22.61 (20.55–26.66)	23.27 (20.55–26.66)
Total Body Calcium (g)	2.47	0.48	1011.5 (798–1201)	955 (861–1068)	996.5 (882–1157)	1017 (882–1201)
Dry Weight (kg)	2.03	0.57	68.55 (55.52–75.6)	63.79 (57.68–78.31)	68.15 (54.49–77.21)	66.89 (54.49–73.64)
Fat-Free Mass Hydration (%)	2.95	0.4	74.27 (72.34–74.54)	75.32 (72.94–77.98)	74.45 (73.04–76.29)	74.51 (73.04–78.03)
Malnutrition Index	4.16	0.24	0.84 (0.78–0.89)	0.85 (0.83–0.91)	0.82 (0.73–0.91)	0.82 (0.74–0.99)

**Table 3:** The post hoc analysis of the levodopa–carbidopa intestinal gel on the bioimpedance measurements after the statistical extrapolation of values. Only values with *p* < 0.05 are presented.

Variable	The Post Hoc Analysis Performed with The Dunn–Bonferroni Test			
	Median (IQR) V0	Median (IQR) V0.5	Median (IQR) V1	Median (IQR) V2
Resting Metabolic Rate (kcal)	1441.5 (1299–1612)		1458 (1276–1606.5)	
Extracellular Water (%)		47.01 (46.50–48.60)	46.08 (43.94–48.13)	
Intracellular Water (%)		18.90 (15.92–23.05)	19.05 (16.24–22.55)	
Extracellular Water/Intracellular Water	0.89 (0.84–0.92)	0.89 (0.87–0.95)	0.85 (0.78–0.93)	

**Table 4:** Impact of the LCIG therapy on the selected clinical measurements and biochemical results (raw data analysis) performed with Friedman’s ANOVA. *p* is considered significant with values < 0.05.

Variable	Friedman’s ANOVA Test Value	*p*-Value	Median (IQR) V0	Median (IQR) V0.5	Median (IQR) V1	Median (IQR) V2
LEDD (mg)	0.17	0.98	1360 (1150–1360)	1688 (1421–1848)	1750 (1159–1750)	1661.50 (1159–2053)
ACE-III	2.88	0.24	76 (69–76)	-	82.50 (72.00–82.50)	69 (64–79)
Beck’s Depression Inventory	0.24	0.89	18 (12–18)	-	18 (10–18)	13 (11–18)
Hauser OFF (h)	13.90	0.003	7.5 (6–7.5)	2 (1–3)	2.5 (1.5–2.5)	2.(0.5–4.25)
Hauser dys (h)	12.63	0.005	4 (2.5–4)	0 (0–1)	0 (0–0)	0 (0–0)
Hauser ON (h)	12.62	0.005	7 (4.5–7)	14 (13–14.5)	12 (12–12)	13.5 (12.2–15)
Weight (kg)	0.57	0.9	68 (56–68)	65 (43–80)	71.25 (56–71.25)	68 (58–82)
Body mass index (kg/m^2^)	2.27	0.52	24.8 (21.5–24.8)	23 (17.7–27.6)	26.20 (19.60–26.20)	22.20 (20–23.50)
Waist circumference (cm)	2.38	0.5	84 (75–97)	82 (67–93)	92 (77–92)	90 (81–110)
Hip circumference (cm)	3.24	0.36	92.5 (85.5–92.5)	85 (80–104)	97 (87–97)	96 (90–102)
Tricep skinfold thickness (mm)	5.16	0.16	12 (8.5–12)	10 (7–15)	9 (8–9)	11 (10–15)
Abdominal skinfold thickness (mm)	0.75	0.86	12 (10–12)	12 (10–22)	14 (10–14)	19.5 (10–29)
Total serum protein (g/dL)	0.86	0.65	6.7 (6.2–6.7)	6.35 (6.15–6.95)	6.90 (6.6–6.9)	-
Albumin (g/dL)	3.26	0.2	4.3 (4.1–4.3)	4 (3.6–4.1)	4.3 (4–4.3)	-
Creatinine (mg/dL)	8.53	0.01	0.69 (0.63–0.69)	0.62 (0.5–0.69)	0.63 (0.54–0.63)	-
Alanine transaminase (U/L)	8.08	0.02	5 (5–5)	6 (5–6)	5 (5–5)	-
Aspartate transaminase (U/L)	0.16	0.92	15 (13–15)	18 (12–22)	15 (13–15)	-
Vitamin B12 (pg/mL)	1.61	0.45	308.5 (243–308.5)	264.5 (215–346)	247.5 (209.5–247.5)	-
Total cholesterol (mg/dL)			183 (170–183)	170 (150–215)	190 (170–190)	-
High-density lipoprotein (mg/dL)			56 (51–56)	55 (45–59)	53(50.5–53)	-
Low-density lipoprotein (mg/dL)	1.43	0.49	112 (94–112)	102 (77–135)	113 (97.5–113)	-
Triglycerides (mg/dL)	0.89	0.64	69 (59–69)	80 (61–156)	86.5 (76.5–86.5)	-

IQR—interquartile range; LEDD—levodopa equivalent daily dose; ACE-III—Addenbrooke’s Cognitive Examination-III.

**Table 5:** The post hoc analysis of the LCIG therapy on the selected clinical measurements and biochemical results (raw data analysis). Only values with *p* < 0.05 are presented.

Variable	The Post Hoc Analysis Performed with The Dunn–Bonferroni Test			
	Median (IQR) V0	Median (IQR) V0.5	Median (IQR) V1	Median (IQR) V2
Hauser OFF (h)	7.5 (6–7.5)	2 (1–3)	2.5 (1.5–2.5)	
Hauser dys (h)	4 (2.5–4)			0 (0–0)
Hauser ON (h)	7 (4.5–7)	14 (13–14.5)	12 (12–12)	13.5 (12.2–15)
Creatinine (mg/dL)	0.69 (0.63–0.69)	0.62 (0.5–0.69)	0.63 (0.54–0.63)	-
Alanine transaminase (U/L)	5 (5–5)		5 (5–5)	-

## Data Availability

The raw data supporting the conclusions of this article will be made available by the authors on request.

## Supplementary Materials

The following supporting information can be downloaded at: https://www.mdpi.com/article/10.3390/jcm14072321/s1, Table S1: Reference ranges set in Maltron Bioscan 920-II Multi-frequency Analyser.

## Abbreviations

The following abbreviations are used in this manuscript:

PD	Parkinson’s Disease
PwP	People with Parkinson’s Disease
LID	Levodopa-induced dyskinesia
LCIG	Levodopa–carbidopa intestinal gel
DBS	Deep brain stimulation
BIA	Bioelectrical impedance analysis
BMI	Body mass index
FAT	Fat mass
FFM	Fat-free mass
BCM	Body cell mass
MI	Malnutrition index
TBW	Total body water
ICW	Intracellular water
ECW	Extracellular water
RMR	Resting metabolic rate
MW	Muscle weight
LEDD	Levodopa equivalent daily dose
ALT	Alanine transaminase
AST	Aspartate transaminase
LDL	Low-density lipoprotein
HDL	High-density lipoprotein
TC	Total cholesterol
TG	Triglycerides
ACE-III	Addenbrooke’s Cognitive Examination-III
