# Supplementary material for: Shaping Outcomes: Levodopa–Carbidopa Intestinal Gel Treatment and Nutrition in Parkinson’s Disease—A Prospective Observational Cohort Study
Source: J Clin Med. 2025 Mar 28;14(7):2321. doi: 10.3390/jcm14072321 (PMC11989982; doi:10.3390/jcm14072321)
Supplement: Supplementary file 1 [file jcm-14-02321-s001.zip › jcm-3509686-supplementary.pdf]

Supplementary Table S1. Reference ranges set in Maltron Bioscan 920-II Multi-frequency Analyser.

**Parameter**	**Male**		**Female**	
	Minimum	Maximum	Minimum	Maximum
Body Cell Mass (kg)	50.0		50.0	
Body Mass Index (kg/m^2)	19.0	30.5	18.5	29.0
Extracellular Water (%)	35.0	48.0	35.0	49.0
Fat (%)	10.0	30.0	18.0	35.0
Total Body Water (%)	69.0	77.0	69.0	77.0
